# Characterization and Genome Structure of Virulent Phage EspM4VN to Control *Enterobacter* sp. M4 Isolated From Plant Soft Rot

**DOI:** 10.3389/fmicb.2020.00885

**Published:** 2020-06-03

**Authors:** Nguyen Cong Thanh, Yuko Nagayoshi, Yasuhiro Fujino, Kazuhiro Iiyama, Naruto Furuya, Yasuaki Hiromasa, Takeo Iwamoto, Katsumi Doi

**Affiliations:** ^1^Microbial Genetics Division, Institute of Genetic Resources, Faculty of Agriculture, Kyushu University, Fukuoka, Japan; ^2^Plant Protection Research Institute, Hanoi, Vietnam; ^3^Laboratory of Plant Pathology, Faculty of Agriculture, Kyushu University, Fukuoka, Japan; ^4^Attached Promotive Center for International Education and Research of Agriculture, Faculty of Agriculture, Kyushu University, Fukuoka, Japan; ^5^Core Research Facilities for Basic Science, Research Center for Medical Sciences, The Jikei University School of Medicine, Tokyo, Japan

**Keywords:** virulent phage, *Enterobacter*, *Agtrevirus*, soft rot disease, genome

## Abstract

*Enterobacter* sp. M4 and other bacterial strains were isolated from plant soft rot disease. Virulent phages such as EspM4VN isolated from soil are trending biological controls for plant disease. This phage has an icosahedral head (100 nm in diameter), a neck, and a contractile sheath (100 nm long and 18 nm wide). It belongs to the Ackermannviridae family and resembles *Shigella* phage Ag3 and *Dickeya* phages JA15 and XF4. We report herein that EspM4VN was stable from 10°C to 50°C and pH 4 to 10 but deactivated at 70°C and pH 3 and 12. This phage formed clear plaques only on *Enterobacter* sp. M4 among tested bacterial strains. A one-step growth curve showed that the latent phase was 20 min, rise period was 10 min, and an average of 122 phage particles were released from each absorbed cell. We found the phage’s genome size was 160,766 bp and that it annotated 219 open reading frames. The genome organization of EspM4VN has high similarity with the *Salmonella* phage SKML-39; *Dickeya* phages Coodle, PP35, JA15, and Limestone; and *Shigella* phage Ag3. The phage EspM4VN has five tRNA species, four tail-spike proteins, and a thymidylate synthase. Phylogenetic analysis based on structural proteins and enzymes indicated that EspM4VN was identified as a member of the genus *Agtrevirus*, subfamily Aglimvirinae, family Ackermannviridae.

## Introduction

Soft rot is a serious disease of worldwide economic significance that occurs on fleshy vegetables such as potato, carrot, eggplant, squash, and tomato and is caused by *Pectobacterium* and *Dickeya* species (formerly known as the soft rot *Erwinia*) (Ma et al., 2007; Masyahit et al., 2009; Thanh et al., 2009; Lim et al., 2013; George et al., 2018; Rossmann et al., 2018). Both bacterial species are in the soft rot Pectobacteriaceae (SRP) family. Recently, *Enterobacter asburiae* was found to cause mulberry wilt disease and soft rot on *Amorphophallus konjac* in China (Wang et al., 2010; Wu et al., 2011). *E. asburiae* is a gram-negative, facultative anaerobic, oxidase negative, non-motile, and non-pigmented rod-shaped species isolated from soil, water, and food products and previously known as an epiphytic bacteria (Lau et al., 2014). In addition, *Enterobacter cloacae* was identified as the cause of an unreported bacterial disease in chili pepper (*Capsicum annuum* L.) (García-González et al., 2018) and bacterial soft rot disease in dragon fruit (*Hylocereus* spp.) (Masyahit et al., 2009). The genus *Enterobacter* is classified in the *Enterobacter*–*Escherichia* clade, which is different from the *Pectobacterium*–*Dickeya* clade of the order Enterobacteriales (Adeolu et al., 2016). The harmful effects of *Enterobacter* species to soft rot have been argued. Kõiv et al. (2015) reported that *Enterobacter* and *Pseudomonas* were the most dominant genera in infected potato tissues.

In Southeast Asian countries such as Vietnam, chemical agents have been a dominant strategy for controlling plant disease. The use of pesticides in the region has been increasing annually and has caused severe issues for human health and the natural environment due to residual concentration in products, land, air, and water. Roughly 10,000 tons of pesticides was used annually in the 1980s, and this number reached 40,000 tons in the early 2000s (Engineering and Consulting Firms Association, 2006). In addition to pesticides, integrated pest management involving cultivation and pest-resistant plants has been applied widely. However, various bacterial organisms have developed pesticide resistance such as kasugamycin resistance tested in *Erwinia* sp. or carbapenem and cephalosporin resistance reported in *Enterobacteriaceae* (McGhee and Sundin, 2011). Despite a growing need for innovative solutions to this problem, the use of biological control agents such as bacteriophages has not been significantly considered.

Bacteriophages (phage, φ) are viruses specific to bacteria. These viruses have been playing an important role in plant disease control, known as phage therapy (Jones et al., 2007; Balogh et al., 2010). More interestingly, no serious side effects have yet been found on human and animal cells (Sulakvelidze and Kutter, 2005; Kusradze et al., 2016). Therefore, phage control may provide a promising solution for environment- and human-friendly treatment of bacteria-related plant disease. However, the utility of phage therapy often presents a challenge because of specificity to their host bacteria. Two broad-host lysates, φPD10.3 and φPD23.1, have been used for effectively controlling the strains of *Pectobacterium* and *Dickeya* spp. (Czajkowski et al., 2015a). Gašić et al. (2018) reported that *Xanthomonas euvesicatoria* phage Kφ1 had potential for biological control against pepper bacterial spot. Additionally, phage typing was also widely used in bacterial characterization and differentiation. For example, it was reported that nine of 22 *Enterobacter* phages investigated were isolated and used for distinguishing *Enterobacter* species (Loessner et al., 1993). Biological control using phages for soft rot has been applied to soft rot *Enterobacteriaceae* (SRE) such as *Pectobacterium carotovorum* (Muturi et al., 2019), *Dickeya solani* (Carstens et al., 2018), and *Pectobacterium atrosepticum* (Buttimer et al., 2018) causing potato soft rot. However, there is no report studying phages infecting *Enterobacter* species such as *E*. *asburiae, E*. *cloacae*, and *Enterobacter intermedium* and related species. Understanding the biological and genetic characteristics of these phages is beneficial for applying phage therapy against emerging SRE infectious diseases.

Therefore, the aim of this study is to isolate and characterize a phage-infecting *Enterobacter* species to be used against soft rot disease in infected crops.

## Materials and Methods

### Bacterial Strains and Growth Conditions

*Acinetobacter baumannii*, *Escherichia coli*, *Dickeya chrysanthemi* pv. *zeae* 511-3 S2, two *Pantoea* strains, six *Enterobacter* strains, and seven *Pectobacterium* strains were used in this study (Table 1). Bacteria samples were collected from vegetables and fruits in Hanoi and Binh Thuân, Vietnam, and each sample was spread on an LB agar. After incubation for 3 days at 37°C, colonies were selected and purified by streaking on fresh plates three times. Type strains were purchased from the Biological Resource Center, NITE (NBRC, Chiba, Japan), and the *Pectobacterium* species were donated by the Laboratory of Plant Pathology, Kyushu University, for references. All bacteria were cultured in LB broth with shaking. Bacterial strains were cultivated at their optimum temperatures. Grown cells were suspended in 30% glycerol and stored at −80°C until use.

**TABLE 1:** Bacterial strains used in this work and their sensitivities against EspM4VN.

Strain	Source	Accession number	Plaque formation	References
*Pantoea dispersa* TL3	Dragon fruit	LC498100	–	This work
*Enterobacter* sp. B3	Cabbage	LC498101	–	This work
*Enterobacter* sp. B5	Cabbage	LC498102	–	This work
*Enterobacter* sp. BC7	Cabbage	LC415135	–	This work
*Enterobacter* sp. M4	Potato	LC415612	+	This work
*Enterobacter* sp. KT	Potato	LC416590	–	This work
*Acinetobacter baumannii* TL5	Dragon fruit	LC423530	–	This work
*Acinetobacter baumannii* NBRC 109757	Urine	Z93435	–	Bouvet and Grimont (1986)
*Acinetobacter junii* NBRC 109759	Urine	Z93438	–	Bouvet and Grimont (1986)
*Enterobacter asburiae* NBRC 109912	Human, lochia exudate	CP011863	–	Hoffmann et al. (2005)
*Escherichia coli* DHα5			–	RBC Bioscience
*Pectobacterium carotovorum* subsp. *carotovora* 489-4	Cabbage		–	Plant Pathology Laboratory, Kyushu University
*Pectobacterium carotovora* subsp. *carotovora* 489-5	Cabbage		–	Plant Pathology Laboratory, Kyushu University
*Pectobacterium carotovora* subsp. *carotovora* 493-1	Potato		–	Plant Pathology Laboratory, Kyushu University
*Pectobacterium carotovora* subsp. *betavasculorum* ATCC 43762^T^	Sugar beet	U80198	–	Gardan et al. (2003)
*Pectobacterium carotovora* subsp. *wasabiae* ATCC 43316^T^	Japanese horseradish	U80199	–	Gardan et al. (2003)
*Pectobacterium carotovora* subsp. *atroseptica* ATCC 33260^T^	Solanum tuberosum	Z96090	–	Gardan et al. (2003)
*Pectobacterium carotovora* subsp. *carotovora* ATCC 15713^T^	Potato	U80197	–	Hauben et al. (1998)
*Pectobacterium chrysanthemi* pv. *Zeae* 511-3 S2	Corn		–	Plant Pathology Laboratory, Kyushu University
*Pectobacterium milletiae* 1			–	National Institute of Agrobiological Sciences, Japan

### Bacterial Identification

Chromosomal DNA of isolates was isolated using MightyPrep reagent for DNA (TaKaRa Bio Inc., Shiga, Japan). 16S rRNA genes of isolated DNAs were amplified by PCR using Tks Gflex DNA polymerase (TaKaRa Bio Inc) and primer sets 27f (5′-AGAGTTTGATCCTGGCTCAG-3′) and 1492r (5′-ACGGCTACCTTGTTACGACCT-3′). PCR products were cloned into the pTA2 vector (Toyobo Co., Ltd., Osaka, Japan) and sequenced using a BigDye Terminator v3.1 cycle sequencing kit (Life Technologies, Carlsbad, CA, United States). Sequences were determined using an Applied Biosystems Gene Analyzer 3130*xl* (Life Technologies). A phylogenetic tree was constructed using the neighbor-joining method with the program of the GENETYX software (GENETYX, Tokyo, Japan).

### Isolation of the Phage

Soil samples were collected from cabbage or potato fields in Bac Ninh province, Vietnam. These samples were suspended with SM buffer (Van Twest and Kropinski, 2009). Soil suspensions were added to a log-phase culture of *Enterobacter* sp. M4 for an enrichment culture. After shaking (37°C, 180 rpm, 24 h), the culture solution was centrifuged (10,000 × *g*, 10 min, 4°C). The supernatant was filtered through a pore size of 0.45 μm (Advantec, Tokyo, Japan). The phage was assayed using the soft agar overlap technique (Adams, 1959). After overnight incubation, typical plaques were suspended in an SM buffer and purified through five rounds of single-plaque isolation.

### Purification of Phage

Phage particles were purified using cesium chloride (CsCl) gradient ultracentrifugation according to a previously reported protocol (Sambrook and Russell, 2001). Briefly, enriched phage lysate (5 × 10^10^ plaque-forming units, pfu) was treated with DNase I (1 μg/ml) and RNase A (1 μg/ml) at 37°C for 1 h. Phage lysates were centrifuged (10,000 × *g*, 10 min, 4°C). The supernatant was mixed with 1 M NaCl and 10% (w/v) polyethylene glycol 8000, and the mixture was stored at 4°C for 2 h. After centrifugation (12,000 × *g*, 30 min, 4°C), the pellets were suspended in 4 ml of TBT buffer (100 mM Tris–HCl at pH 7.5, 100 mM NaCl, and 10 mM MgCl_2_). The resulting suspension was gently mixed with 4.5 g of CsCl and loaded onto a step-layered CsCl gradient (1 ml of 0.5 g/ml CsCl, 2.5 ml of 0.775 g/ml CsCl, and 2.5 ml of 1 g/ml CsCl) prepared in a 38.5-ml Ultra-Clear tube (Beckman Coulter, Indianapolis, IN, United States). The gradient was centrifuged at 22,000 rpm in the swinging-bucket SW28 rotor for 2 h at 4°C in Optima XE-90 ultracentrifuge (Beckmann Coulter). The phage band was dialyzed against dialysis buffer (10 mM NaCl, 50 mM Tris–HCl at pH 8.0, and 10 mM MgCl_2_) for 4 h. Purified phage particles were resuspended in SM buffer with 25% glycerol and then stored at −80°C.

### Transmission Electron Microscopy

Phage morphology was determined using transmission electron microscopy (TEM) to observe negatively stained preparations (Luo et al., 2012). Phage particles picked up from a purified single plaque were suspended to 2% (wt/vol) phosphotungstic acid (pH 7.2) and then applied to the surface of a glow-discharged carbon/formvar-coated grid (200-mesh copper grid). Negatively stained phage particles were examined using a Hitachi H-7500 transmission electron microscope operated at 80 kV (Hitachi High-Technologies Corp., Tokyo, Japan). The phage size was determined from at least 10 measurements.

### Host Range Test

The determination of host range was followed by Addy’s description with minor modifications (Addy et al., 2019). Briefly, 10 μl of serial dilutions of phage solution (10^8^ pfu/ml) was dropped on double-layer agar plates. The top layer was prepared with 100 μl of each strain (OD_660_ of 0.4) mixed with 7 ml of LB. The plates were incubated overnight at bacterial optimum temperature. The phage sensitivity of the bacteria was confirmed by observation of clear zones or plaques. And then further experiment was performed with plaque essay using the dilutions to confirm if they were affected by the phage.

### Determination of the Optimal MOI

Optimal multiplicity of infection (MOI) was determined according to the report of Czajkowski et al. (2015a). The indicator strain (4 × 10^8^ colony-forming units, cfu/ml) at the early log phase was infected with phage at different MOIs of 0.1, 1, and 10. After a 15-min incubation at 37°C, the mixtures were centrifuged (12,000 × *g*, 10 min, room temperature). Supernatants were then filtered through a 0.45-μm pore size filter and titrated as described above. The highest titer was considered the optimal MOI. All experiments were averaged from results of triplicate experiments.

### Thermal and pH Stability

Phage stability was carried out as described in our previous report (Luo et al., 2012). Briefly, to examine thermal stability, phage stocks (1.0 × 10^7^ pfu/ml in SM buffer) were incubated separately for 60 min at temperature ranges of 10–60°C. For pH stability, 1.0 × 10^7^ pfu/ml of phage suspension was added to an SM buffer at different pHs (pHs 3–11) and then incubated at 25°C for 24 h. After incubation, the surviving phages were enumerated by the double-layer agar method. All tests were performed in triplicate.

### One-Step Growth Curve

One-step growth experiments were performed to determine the latent period and burst size as described previously (Nagayoshi et al., 2016; Addy et al., 2019). *Enterobacter* sp. M4 cells were harvested by centrifugation of the early-log-phase culture (4 × 10^8^ cfu/ml) and resuspended in LB broth. Phage was added at an MOI of 1 and allowed to adsorb for 15 min at 37°C. Cell pellet was washed with 1 ml of fresh LB broth three times. The resuspension was added to 100 ml of LB broth and cultured at 37°C. Samples were taken at 10-min intervals (up to 120 min), and phage titers were then determined by the double-layer agar plate method to obtain one-step curves. The average burst size of the phage was calculated according to the report of Bolger-Munro et al. (2013).

### Stability With Surfactants

Phage stability was tested with various concentrations of five surfactants: Tween 20, sodium dodecyl sulfate (SDS), ethanol, skim milk (Becton, Dickinson, and Company, Franklin Lakes, NJ, United States), and sucrose. Tween 20 and SDS were chosen as a non-ionic detergent and anionic surfactant, respectively. Ethanol was examined as a standard disinfectant. Phage particles were suspended in each solution at various concentrations for 2 h at room temperature. The resistant capability was investigated by plaque assay. Experiments were performed in triplicate.

### Phage DNA Extraction and Genome Analysis

Purified phage particles were used for phage DNA extraction with TE saturated phenol (pH 8.0) (Nippon Gene Co., Ltd., Tokyo, Japan) and phenol–chloroform and were precipitated with sodium acetate and ethanol. The precipitate was washed twice with 70% ethanol, air-dried, and resuspended in the TE buffer.

Phage whole genome was sequenced using the PacBio RSII platform (Pacific Biosciences of California, Inc., Menlo Park, CA, United States). The filtered reads were assembled using HGAP version 2.3.0 (Chin et al., 2013) and resulted in a one-contig scaffold. Potential open reading frames (ORFs) were predicted by the Microbial Genome Annotation Pipeline (MiGAP^1^). The BLAST algorithm at the National Center of Biotechnology Information (NCBI) was used to search for similarities. Analysis of the nucleotide sequences was performed with Geneious Prime (Biomatters Ltd., Auckland, New Zealand). The search for tRNA genes was performed with tRNAscan-SE (Schattner et al., 2005) and ARAGORN ver. 1.2.38 (Laslett and Canback, 2004). Phylogenetic analysis was performed with ClustalW ver. 2.1^2^, and phylogenetic trees were generated using the neighbor-joining method. Prediction of promoter regions was carried out with Neural Network Promoter Prediction^3^.

### Structural Protein Identification by Mass Spectroscopy

To analyze virion proteins, phage particles purified by ultracentrifugation were mixed with lysis buffer (62.5 mM Tris–HCl, pH 6.8, containing 5% 2-mercaptoethanol, 2% SDS, 10% glycerol, and 0.01% bromophenol blue) and boiled for 10 min. Prepared proteins were then separated by electrophoresis on precast SDS 15% polyacrylamide gels (e-PAGEL E-T/R15L, ATTO Corporation, Tokyo, Japan).

### In-Gel Digestion

Excised gel pieces were destained with 100 μl of 50% acetonitrile containing 25 mM ammonium bicarbonate solution for 1 h at room temperature with gentle agitation. Destained gel pieces were treated for reduction and alkylation using 100 μl of 10 mM DTT in 25 mm ammonium bicarbonate for 45 min at 56°C and 100 μl of freshly prepared 10 mM iodoacetamide in 25 mM ammonium bicarbonate in the dark for 30 min at 37°C. After gel plugs were dried, 400 ng of sequencing-grade trypsin (Trypsin Gold, Promega, Madison, WI, United States) in 20 μl of 25 mM ammonium bicarbonate was added and incubated for 12 h at 37°C. Digested peptides were recovered from the gel plugs using 50 μl of 50% acetonitrile in 5% formic acid (FA) for 30 min at 25°C. The extracted peptides were concentrated in a speed vacuum concentrator and added to 20 μl of 5% acetonitrile in 0.1% FA.

### Tandem Mass Spectrometry-Based Proteomics

A Nano-HPLC system (nanoADVANCE, Bruker-Michrom, Billerica, MA, United States) was used to identify proteins automatically using a micro-column switching device coupled to an autosampler and a nanogradient generator. Peptide solution (5 μl) was loaded onto a C18 reversed-phase capillary column (100 μm ID × 30 cm, Zaplous αPep C18; AMR, Tokyo, Japan) in conjunction with a Magic AQ C18 trapping column (300 μm ID × 10 mm; Bruker-Michrom). The peptides were separated using a nanoflow linear acetonitrile gradient of buffer A (0.1% FA) and buffer B (0.1% FA, 99.9% acetonitrile), going from 5 to 45% buffer B over 50 min at a flow rate of 500 nl/min. The column was then washed in 95% buffer B for 5 min. Hystar 3.2 system control software (Bruker Daltonics Inc., Billerica, MA, United States) was used to control the entire process. The eluted peptides were ionized through a CaptiveSpray source (Bruker Daltonics) and introduced into a Maxis 3G Q-TOF mass spectrometer (Bruker Daltonics) set up in a data-dependent MS/MS mode to acquire full scans (*m*/*z* acquisition range from 50 to 2,200 Da). The four most intense peaks in any full scan were selected as precursor ions and fragmented using collision energy. MS/MS spectra were interpreted, and peak lists were generated using DataAnalysis 4.1 and BioTools 3.2.

### Protein Identification

The filtered data were searched on the Mascot 2.2 server (Matrix Science) using the NCBInr (NCBI 201805) database and custom expected protein databases for EspM4VN. Fixed modification was set on cysteine with carbamidomethylation. Variable modification was based on methionine with oxidation and asparagine/glutamine with deamidation. Maximum missed cleavage was set to two and limited to trypsin cleavage sites. Precursor mass tolerance (MS) and fragment mass tolerance (MS/MS) were set to 100 ppm and ± 0.6 Da, respectively. Positive protein identifications using a threshold of 0.05 were used. Peptides scoring <20 were automatically rejected, ensuring all protein identifications were based on reliable peptide identifications. Protein identification was set to require at least two unique peptides. Homology searches against matched protein sequences were conducted using the BLASTp program^4^.

### Accession Number

The whole-genome sequence of phage EspM4VN was submitted to DDBJ under accession number LC373201. The 16S rDNA sequences of the isolates have been deposited in the DDBJ under accession numbers LC415135, LC415612, LC416590, LC423530, LC498100, LC498101, and LC498102.

## Results

### Identification of Isolated Bacteria

The bacteria used in this study were isolated from diseased plants including potato, cabbage, and dragon fruit (Table 1). After colony purification, isolated bacteria were applied to 16S rDNA sequence analysis. The phylogenetic analysis from sequence data showed that the isolated strains B3, B5, and M4 belonged to the *Enterobacter* cluster (Supplementary Figure S1). Other isolates, TL3 and TL5, were identified as *Pantoea dispersa* and *A. baumannii*, respectively. Strains BC7 and KT were positioned at another *Enterobacteriaceae* cluster.

### Isolation and Morphology of EspM4VN

**FIGURE 1:**
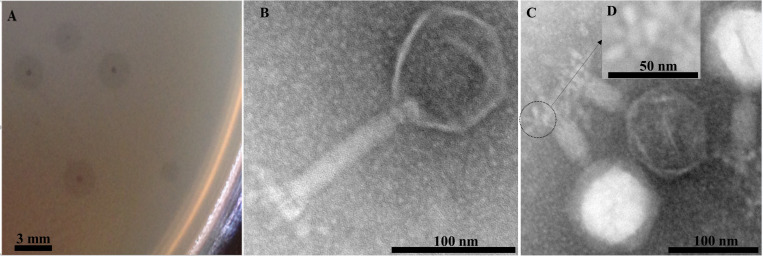
Plaques formed in the top agar layer **(A)**, morphology of the extended tail phage EspM4VN **(B)**, contracted tail phage EspM4VN **(C)**, and displaying an umbrella-like structure **(D)**. Bars indicate 3 mm **(A)**, 100 nm **(B,C)**, and 50 nm **(D)**.

EspM4VN formed large semi-turbid plaques on a lawn of the strain M4. The plaques were approximately 0.5 mm in diameter with a halo effect (2.5 mm) (Figure 1A). Purified phage particles from the plaques were applied to TEM analysis. The phage particles were composed of an icosahedral-shaped head approximately 100 nm in diameter, a neck, and a contractile sheath 100 nm in length and 18 nm in width (Figure 1B). Intermediate structures with both prongs and stars appeared as have been observed in some Ackermannviridae phages such as *Agtrevirus* Ag3, *Limestonevirus* JA15, and XF4 (Anany et al., 2011; Day et al., 2017). Thus, the phage morphology of EspM4VN is that of the genus *Agtrevirus* belonging to the Ackermannviridae family according to ICTV rule 3.12 (Adriaenssens et al., 2018).

### Host Range of EspM4VN4

The host range of EspM4VN was determined with six strains of *Enterobacter*, nine strains of *Pectobacterium*, two *Acinetobacter* species, *A. baumannii* TL5, *P. dispersa* TL3, and *E. coli* DHα5 (Table 1). EspM4VN formed clear plaques only on *Enterobacter* strain M4. A phage suspension of 4 × 10^6^ pfu/ml was applied to strain M4 at a low density level for a similar host range experiment used by others (Adriaenssens et al., 2012). It showed a quite narrow host range even in the same *Enterobacter* cluster.

### Thermal and pH Stability of EspM4VN

**FIGURE 2:**
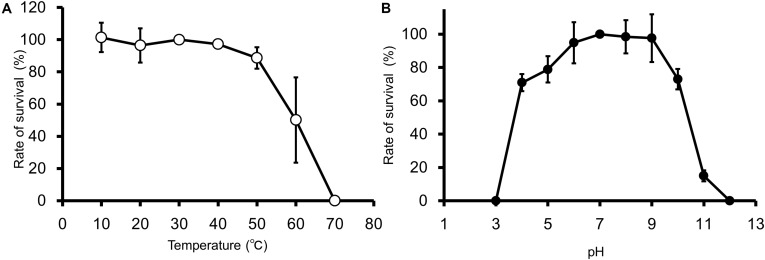
Thermal and pH stability of EspM4VN. Course of survival ratio (%) of EspM4VN particles during heat **(A)** and pH **(B)** treatments is plotted. The mean titer is shown from triplicate assays.

EspM4VN kept stable infectivity at temperatures 10–50°C (Figure 2A) and pH 4–10 (Figure 2B). The phage was deactivated at 70°C and pHs 3 and 12. Related phages infecting *Dickeya* strains were reported to be stable at temperatures from 4°C to 37°C but not stable at 50°C for 24 h, and they showed stability at pHs 5–12 (Czajkowski et al., 2014).

### One-Step Growth of EspM4VN

**FIGURE 3:**
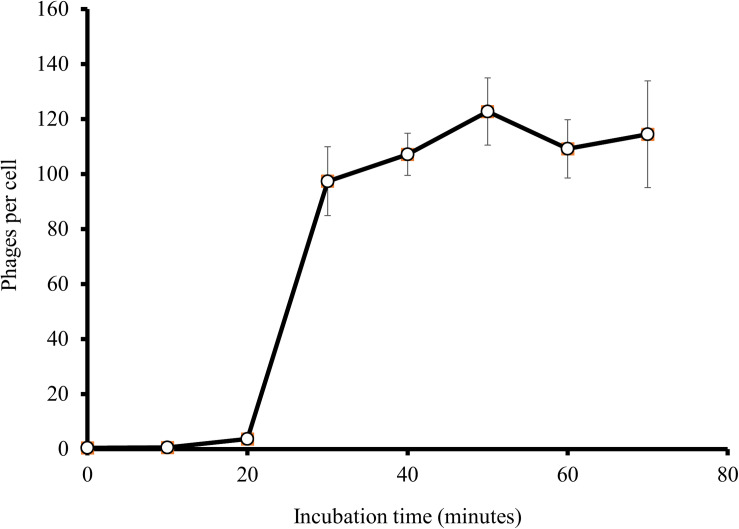
One-step growth curve of the EspM4VN.

At an MOI of 1, a one-step growth curve showed that the latent phase of EspM4VN was 20 min, the rise period was 10 min, and an average of 122 phage particles were released from each absorbed cell (Figure 3). The latent phase of EspM4VN was much shorter than that of LIMEstone1 and LIMEstone2 (60 and 65 min, respectively) or soilborne lytic phages (40 min) infecting *Dickeya* strains (Adriaenssens et al., 2012; Czajkowski et al., 2014).

### Stability of EspM4VN Within Various Chemicals

**FIGURE 4:**
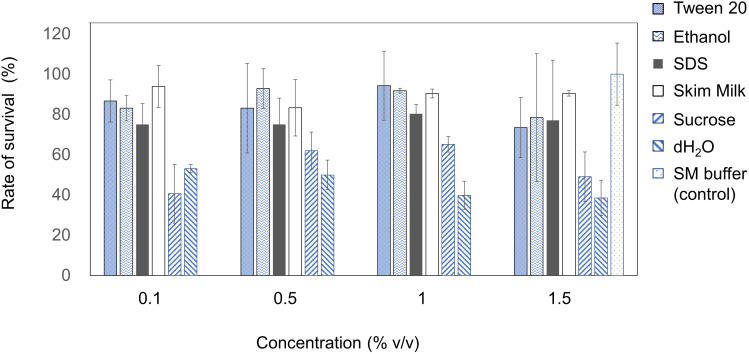
Chemical sensitivity of EspM4VN. Phage particles were inoculated in LB containing different concentrations of Tween 20, ethanol, SDS, skim milk, and sucrose from 0.1 to 1.5?%. SM buffer (outlined bars) was used as a control. Quantification was achieved by plaque assay. The plates were incubated at 37°C for 12 h, and the phage growth was assayed by plaque-forming units (pfu). Experiments were performed in triplicate on three different occasions, and means ± SD are shown.

The stability of EspM4VN was analyzed against detergents and surfactants (Figure 4). Phage viability in sterile water was approximately 50% or less compared with that in SM buffer. Up to 1.5% (v/v) Tween 20 and ethanol did not inhibit phage infection. By contrast, the phage showed resistance (>70%) to 0.1 to 1.5% SDS. EspM4VN was stable in skim milk at all concentrations examined.

### Genomic Analysis of EspM4VN

**FIGURE 5:**
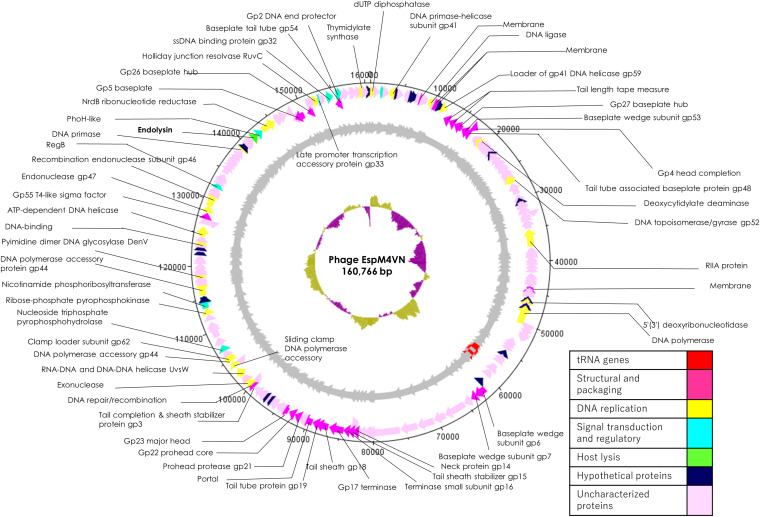
Genome map of EspM4VN. The outer lane represents genes in the plus strand. The next lane illustrates genes on the minus strand. The lane with black peaks and valleys indicate the GC content, while the innermost lane (green and violet) shows a GC skew analysis.

**FIGURE 6:**
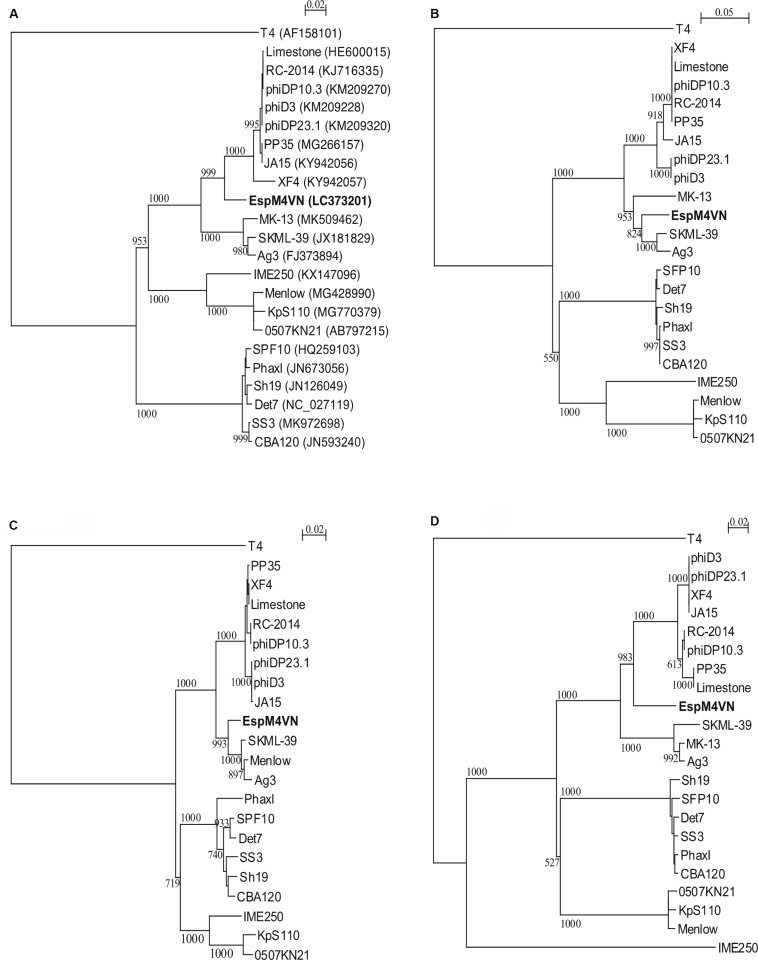
Phylogenetic trees generated based on **(A)** DNA polymerases, **(B)** DNA ligase, **(C)** major capsid protein, and **(D)** baseplate hub subunit of EspM4VN and homologous proteins from other phage members of the Ackermannviridae family. Nucleic acid sequences were compared using ClustalW, and phylogenetic trees were generated using the neighbor-joining method. Numbers in brackets show the gene ID.

Nucleotide sequence analysis of the EspM4VN showed the genome size to be 160,766 bp with a G + C content of 50.7% and that 219 ORFs were annotated (Figure 5 and Supplementary Table S1). BLAST search revealed that the EspM4VN genome showed high similarity to *Agtrevirus* SKML-39 (JX181829; identity 95.3%), *Limestonevirus*, Coodle (MH807820; 95.2%), PP35 (MG266157; 95.2%), JA15 (KY942056; 95.1%), LIMEstone1 (93%), phiDP23.1 (93%), φD3 (93%), and *Agtrevirus* Ag3 (90%) (Supplementary Table S2). EspM4VN as a Ackermannviridae infects *Enterobacter*, and its genome has significant homology with other *Enterobacter* phages such as myPSH1140 (MG999954; Manohar et al., 2019), CC31 (GU323318; Petrov et al., 2010), and vB_EaeM_φEap-3 (KT321315; Zhao et al., 2019). Analysis demonstrated that EspM4VN tRNA species and their codons were as follows: tRNA^*Ser*^ (TCA), tRNA^*Asn*^ (AAC), tRNA^*Tyr*^ (TAC), tRNA^*Ser*^ (AGC), and tRNA^*Asp*^ (GAT). Two unique regulatory motifs, TTCAAT[N_14_]TATAAT and CTAAATAcCcc, were found in their entireties in the EspM4VN genome (positions 7219–7244, 143195–143220, 152828–152853, 158832–158857, 61226–61251, and 65377–65387, 70604–70614, 98591–98601, 101772–101782, and 53611–53621). Phylogenetic trees of structural proteins (major capsid protein and baseplate) and enzymes (DNA polymerase and ligase) showed that EspM4VN formed an independent cluster among Ackermannviridae phages (Figure 6). Based on the phylogenetic analysis of ligase and major capsid protein, EspM4VN was positioned in the same cluster of *Agtrevirus*, *Shigella* phage Ag3, and *Salmonella* phage SKML-39 and not in the same cluster of *Limestonevirus* and *Kuttervirus* belonging to Ackermannviridae (Figures 6B,C). The baseplate protein of EspM4VN was not positioned in *Agtrevirus* but in *Limestonevirus* (Figure 6D).

### Protein Analysis of EspM4VN

**FIGURE 7:**
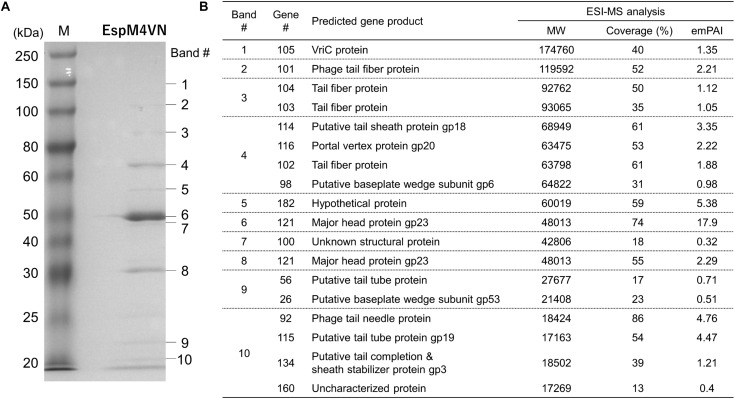
Bacteriophage EspM4VN structural proteins separated in SDS-PAGE gel **(A)** and identification of structural proteins with ESI-MS/MS **(B)**. **(A)** For SDS-PAGE, EspM4VN particles (∼10^12^ pfu) were mixed with lysis buffer and then boiled for 10 min. Phage proteins were separated in a 15% acrylamide SDS-PAGE gel for approximately 19 h at 50 V at 22°C. The bands were stained with Coomassie Brilliant Blue according to the protocol provided by the manufacturer. For ESI-MS/MS analysis of phage structural proteins, protein bands obtained from SDS-PAGE were excised from the gel with a sterile scalpel.

For identification and further characterization of bacteriophage EspM4VN protein, we performed SDS-PAGE and MS analyses of the purified phage. Ten major protein bands were found in the gel, and band patterns including a major band around 50 kDa (band 6 in Figure 7) were similar to those of *Dickeya* spp. bacteriophage (Czajkowski et al., 2015b), *Shigella boydii* phage Ag3 (Anany et al., 2011), and broad-host-range phages φPD23.1 and φD10.3 (Czajkowski et al., 2015a). Ten protein bands excised from the SDS-PAGE gel were digested by trypsin. Proteomic analysis was performed using a quadrupole time-of-flight tandem mass spectrometer. Peptides were identified by matching the identified peaks to the predicted protein sequence library of the custom EspM4VN gene including 219 ORFs or the NCBInr virus database using the in-house Mascot server. In all 10 gel samples, proteins matched against EspM4VN proteins showed much higher Mascot scores than other bacteriophage proteins including *Dickeya* and *Shigella*, indicating that the purified bacteriophage was from the EspM4VN gene.

A total of 18 EspM4VN proteins were identified and are listed in Figure 7 along with the gene number, calculated molecular mass, corresponding protein sequence coverage, and predicted annotation by BLASTp protein similarity search. Because 18 identified protein bands were excised from stained bands, these proteins were suggested to be highly expressed proteins. Of these, 16 were identified as structural proteins and two were classified as uncharacterized or unknown proteins. Genes 105 (VriC), 114 (gp18), 116 (gp20), and 121 (gp23) were identified from *Dickeya* phage φD10.3 (Czajkowski et al., 2015a).

Some genes from identified proteins were sequentially located in the genome sequence including genes 25–26, 101–104, and 114–115. BLASTp search indicated that proteins of genes 25–26 were both baseplate subunits and those of 101–104 were all tail-spike-related proteins. Proteins from genes 114–115 were also identified as tail proteins. These proteins were multimeric proteins for function, and all required high expression levels. Analysis of the EspM4VN genome sequence suggested that similar functions or structural proteins were clustered at certain regions as is the case in other phages.

Besides protein identification, Mascot analysis also provides label-free, relative quantitation of proteins, the exponentially modified protein abundance index (emPAI) in a mixture based on protein coverage by the peptide matches in a database search result (Arike and Peil, 2014). The protein of gene 121 yielded the highest emPAI values at gels #6 and #7 (emPAI: 17.9 and 11.2, respectively) and was identified as a major capsid protein, gp23. The gp23 protein nicely migrated at 50 kDa in the gel according to the calculated molecular weight and showed the highest density of the Coomassie Brilliant Blue stain. Other SDS-PAGE analyses showed that the migrations and band densities of EspM4VN were highly similar to those of the *Dickeya* phage φD10.3 sample (Czajkowski et al., 2015a).

Tryptic fragments from the products of genes 92, 100, 114, and 116 included plausible N-terminal sequences (Supplementary Table S3). MS/MS analysis showed that all of the peptides started from the amino acid following Met (next to Met) because N-terminal Met could be co-translationally cleaved by methionine aminopeptidase. The gene 116 product included not only N-terminal end Ala^2^ but also a C-terminal end Glu^563^. The calculated molecular weight of this protein (63.23 kDa without Met^1^) closely matched with band migration (65 kDa) and was identified as a portal vertex protein, gp20.

Among the plausible 219 genes of EspM4VN, gene analysis indicated that 18 proteins did not start from Met at the N-terminal end but Val or Leu. This suggests that EspM4VN uses only the prokaryote expression system as the start codon in the eukaryote expression system is almost exclusively Met (AUG). Gene 115 was found in the gel band #10 sample and included Val as its plausible N-terminal sequence. The expected N-terminal tryptic peptide VTVNFPAFVAGSDTIR was detected seven times via MS/MS with a high Mascot score of 80. In the genome DNA sequence between the stop codon (TGA) of gene 114 and the start codon (GTG) of gene 115, there were no possible Met (AUG) codons found. Based on DNA sequence analysis and MS/MS analysis, we concluded that gene 115 was expressed from Val at the N-terminal amino acid (Supplementary Figure S2). BLASTp analysis and quantification analysis showed that gene 115 was identified as a tail tube protein, gp19, at high expression because its emPAI was 4.5.

**FIGURE 8:**
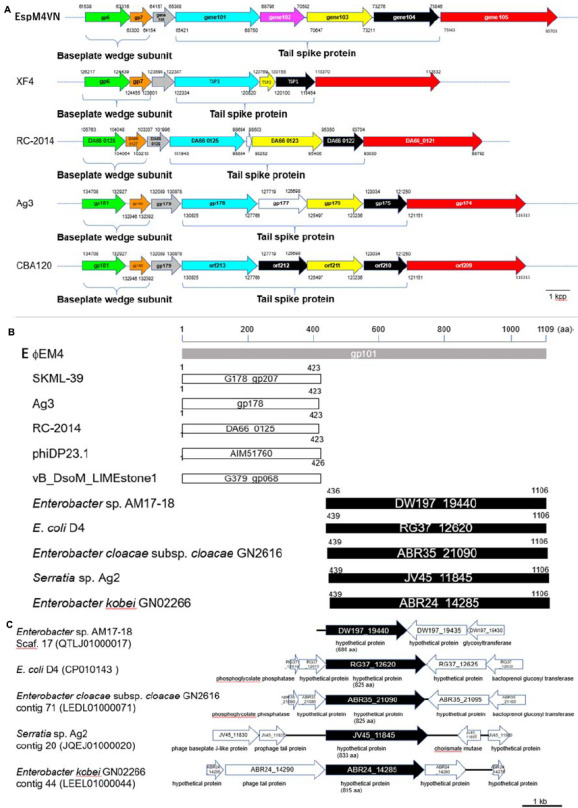
Comparison of gene organization between tail-protein encoding genes in EspM4VN and its homologous genes. **(A)** Gene clusters of phage tail proteins in EspM4VN and Ackermannviridae family phages. Same-colored arrows indicate homologous genes. Blanked arrows show no homology with gene 102 in EspM4VN. Numbers indicate positions of ORFs in the genome. **(B)** Homologous amino acid sequence distribution of gene 101 product (gray bar). White and black bars show partial regions of phage gene products and bacterial chromosomal gene products, respectively. Numbers indicate amino acid positions in each protein. **(C)** Gene organization adjacent to gene 101 homologs in bacterial chromosomes. Black arrows indicate gene 101 homologous genes. Predicted gene products are shown below each arrow.

Gene 101 was found at gel band #2 and had the highest emPAI (2.21) in the gel sample. The molecular weight of gene 101 contained 119,592 Da including 1,109 amino acids. Of the sequence, 52% from the N-terminus to the C-terminus was observed by MS/MS analysis. BLASTp analysis suggested that gene 101 has two unique domains: N-terminal and C-terminal structures. The N-terminal region (1–413) was closely related to other bacteriophage tail proteins (Figure 8B). Interestingly, the C-terminal region (436–1109) was similar to a hypothetical protein from bacteria including *Enterobacter* (WP_117582064.1) or *E. coli* (APK16060.1) (Figure 8C). BLASTp analysis showed that genes 101, 103, and 104 had a pectin lyase fold motif, a typical motif in tail-spike proteins, including the beta helix structure. Gene 101 has a conserved identical sequence region (L^371^-D-X-K-T-V-I-Y-D^379^) with gene 102 (42–50) and gene 103 (42–50). Gene 104 has a part of the identical sequence (V^46^-I-Y-D^49^) and has a conserved motif of the tail spike (N-terminal domain at 97–153).

## Discussion

Bacterial soft rot is one of the most common diseases of important crops in the world. Therefore, several control strategies including physical methods and chemical treatments using copper sulfate, dimethylammonium chloride, sodium hypochlorite, formaldehyde, and antibiotics have been applied. However, these approaches have the risk of increasing environmental burden and health hazards (Berg, 2001; Dasgupta et al., 2007; Pham et al., 2011; Azaiez et al., 2018). The genus *Enterobacter* contains 14 species and two subspecies (Zhu et al., 2017). Among these, the *E. cloacae* complex (Ecc) including *E. cloacae*, *E. asburiae*, *Enterobacter hormaechei*, *Enterobacter kobei*, *Enterobacter ludwigii*, and *Enterobacter nimipressuralis* has genomic heterogeneity and is therefore difficult to identify (Paauw et al., 2008). In this study, we isolated soft rot disease pathogenic bacteria for usage as phage hosts. Phylogenetic analysis of the isolates with their 16S rDNA sequences showed that they located in the Ecc cluster but had unconfirmed identification in the species level. We identified the whole-genome sequence of the strain M4 via *in silico* DNA–DNA hybridization (data not shown). Therefore, the host strain of phage EspM4VN should be *Enterobacter* sp. strain M4, which would be the novel species in the *Enterobacter* genus. It is not enough to prove that isolated strains, including the M4 strain, are the main villain for soft rot disease; therefore, we will examine the function of the strains for maceration with both *in vitro* and in-filed experiments. If so, phage therapy with phage EspM4VN might be a potential solution for soft rot disease.

With morphological observation, EspM4VN belongs to the Ackermannviridae family and resembles *Dickeya* phages φXF4 (Day et al., 2017), but it does not possess short tail spikes as observed in φXF4. Instead, of the gene cluster encoding short-tail-spike proteins of φXF4 (TSP genes), four genes (genes 101–104) consisted of the gene cluster for tail-spike proteins in EspM4VN (Figure 8A). The difference in gene organization may cause morphological differentiation of tail structures between EspM4VN and φXF4. In other words, EspM4VN tails with unfolded tail entities, displaying an umbrella-like structure (Figure 1D). Based on alignment of thymidylate synthase genes, EspM4VN possess the thymidylate synthase gene, a homolog of the deoxyuridylate hydroxymethyltransferase gene of *Escherichia* phage vB_EcoM Sa157lw (AYC62411) with 71.43% homology.

Phage therapy had been increasingly researched and applied as biological control agents against plant pathogens since the early 1920s (Gill and Abedon, 2003; Jones et al., 2007; Balogh et al., 2010). While the application of phages to an integrated plant disease management strategy is relatively simple, cheap, and effective, phage efficacy is highly dependent on general environmental factors and the susceptibility of the target bacterium. Symptomized soft rot disease is a soilborne infection; hence, we isolated phages from farmland, where the above-mentioned plants have been cultivated. In this study, EspM4VN isolated from soil samples was capable of inhibiting only *Enterobacter* strain M4, indicating that EspM4VN has a narrow host range. This is quite similar to other phages such as WS-EP 19, WS-EP 13, WS-EP 20, WS-EP 26, WS-EP 28, WS-EP 57, WS-EP 32, and WS-EP 94, which infect specific *Enterobacter* strains isolated from milk powder and other foods (Loessner et al., 1993). Therefore, EspM4VN may be used in phage cocktails and classification as phage typing for isolated *Enterobacter* strains. One of the reasons for its restricted host range is the characteristic tail structure of the EspM4VN (Figures 1B, 8). One-step growth curve demonstrated that EspM4VN has a short latent phase and quickly reaches burst size. This phage could be a potential epidemic inhibitor for immediate treatment after an outbreak of plant disease. Furthermore, thermal and pH stability tests indicate that it may be applied in tropical and subtropical zones. In the production industry, pesticides are normally formulated in the presence of surface active agents, which can change pesticide adsorption in the soil–water system. Addition of Tween 20 at low and high concentrations increased the adsorption of diazinon and atrazine into soil (Iglesias-Jiménez et al., 1996). Phage EspM4VN resisted Tween 20, ethanol, SDS, and sucrose at various concentrations as well. Skim milk is one of the best substances for product formulation and long-term storage. In addition to some chemicals used in this study, various materials to extend the life of the phage following exposure to various physical factors may be examined (Jones et al., 2007).

Whole-genome analysis of EspM4VN revealed that five tRNAs (Ser1, Ser2, Tyr, Asn, and Asp) were identified. Those tRNAs may correspond to the short latent phage and large burst size because of their positive influences on reproduction in host cells. However, the homologous phage genome *S. boydii* phage Ag3 had four similar tRNAs and a 52-min latent phase. This contrast may be explained by ampleness of the tRNAs presented in the hosts. In this case, the tRNAs of the phage would have less of an effect on the phage environment during infection (Bailly-Bechet et al., 2007).

Like other phage genome sequences, similar functions or structural proteins were clustered at certain regions in the EspM4VN genome. These results suggested that gene locations were closely related with protein expression. However, genes and gene products of EspM4VN exhibit distinctive features (Figure 8). Genes 101 to 104 comprised a gene cluster for tail-spike proteins. Similar cluster structures comprising four genes were observed in *Agtrevirus* (Ag3) and *Limestonevirus* (RC-2014) genomes (Figure 8A). In the genome of *Dickeya* phage φXF4 (taxid: 1983656), whose tail-spike structure is similar to that of EspM4VN, there are three genes in the tail-spike protein cluster. Phage XF4 has a gene structure in which three tail-spike genes (TSP1–TSP3) have been sequentially located at the region (118454–122334) (Day et al., 2017). TSP1, TSP2, and TSP3 have sequence similarities with those of genes 104, 103, and 101. In the gene cluster of the tail-spike protein of *Escherichia* virus CBA120 (taxid: 1987159), the TSP1 gene (*orf213*) and TSP3 gene (*orf211*) show significant homology with genes 101 and 103 (Figure 8A). TSP2 and TSP4 of GBA120 have partial homologies with the product of gene 104. CBA120-TSP2 binds its substrate and functions to recognize the host receptor (Plattner et al., 2019). These findings suggest that EspM4VN tail-spike proteins might form a heteromeric structure in the phage. Only the product of gene 102 shows high homology with the hypothetical proteins of *Enterobacter mori* (WP_157929994.1), *E. coli* (OJN39208.1), *E. cloacae* (WP_063860747.1), and *Klebsiella aerogenes* (WP_063963681.1). Therefore, the unique structure of the product of gene 102 might be caused by the narrow host range of EspM4VN and the possibility that some gene origin of EspM4VN could be from bacterial chromosomes.

Also, gene 101 might be fused with phage-derived gene and bacterial genome-derived gene (Figures 8B,C). Homology analysis of the gene 101 product indicates that its N-terminal region closely resembles a right-handed beta helix region of phage proteins SKML-39 (identity: 94%, 1–423 aa), Ag3 (identity: 94%, 1–423 aa), RC-2014 (identity: 90%, 1–423 aa), phiDP23.1 (identity: 89%, 1–423 aa), and LIMEstone1 (identity: 84%, 1–426 aa) (Figure 8B). The C-terminal region of the gene 101 product has high homology with enterobacterial gene products described as follows: *Enterobacter* sp. AM17-18 (identity: 57%, 436–1106 aa), *E. coli* D4 (identity: 56%, 439–1106 aa), *E. cloacae* ssp. *cloacae* GN2616 (identity: 56%, 439–1106 aa), *Serratia* sp. Ag2 (identity: 38%, 439–1106 aa), and *E. kobei* GN02266 (identity: 36%, 439–1106 aa) (Figure 8B). From gene locations in their chromosome, some of the homologous genes might be inserted in the glycan metabolic, cell wall, and amino acid biosynthesis pathways in *Enterobacter* sp. AM17-18, *E. coli* D4, and *E. cloacae* ssp. *cloacae* GN2616 (Figure 8C). Matilla et al. (2014) suggested that φXF1, φXF3, and φXF4 are very efficient generalized transducers capable of transducing chromosomal markers at high frequencies. The above-mentioned heterogeneous gene structure is reasonable for phage function, as corresponding genes in *Serratia* sp. Ag2 and *E. kobei* GN02266 genomes locate within phage tail gene clusters. This suggests that genes 101 and 102 of EspM4VN existed partially from lysogenic phage genomes or were fused after phage transduction. However, the question remains as to why a tail-spike protein, which is a necessary accessory protein, would start from a rare start codon and whether transcripts longer than 10 kb could be polycistronically synthesized. These and other genetic regulation characteristics of EspM4VN transcription are topics for future studies. We did not observe any lysogenic-related genes such as *int*, *xis*, and *xerD* in the EspM4VN genome; this is a beneficial property of the phage for biocontrol of the plant pathogenic bacteria (Álvarez et al., 2019).

## Data Availability Statement

The whole genome sequence of phage EspM4VN was submitted to DDBJ under accession number LC373201. The 16S rDNA sequences of the isolates have been deposited in the DDBJ under accession numbers to LC415135, LC415612, LC416590, LC423530, LC498100, LC498101, and LC498102.

## Author Contributions

The work presented here was carried out in collaboration between all authors. KD defined the research theme. NT, YN, YF, and KD designed the methods and experiments, carried out the molecular biological and virological experiments, analyzed the data, interpreted the results, and wrote the manuscript. NT, KI, and NF engaged in the experiments using plant pathogens. YH and TI codesigned the protein analysis and ESI-MS/MS experiments and discussed the analyses, interpretation, and presentation. All authors have contributed to, saw, and approved the manuscript.

## Conflict of Interest

The authors declare that the research was conducted in the absence of any commercial or financial relationships that could be construed as a potential conflict of interest.

## Footnotes

1 http://www.migap.org/

2 https://clustalw.ddbj.nig.ac.jp

3 https://www.fruitfly.org/seq_tools/promoter.html

4 http://www.ncbi.nlm.nih.gov/

## Supplementary Material

FIGURE S1 Phylogenetic tree of isolated strains. Their nucleotide sequences were compared using ClustalW, and phylogenetic trees were generated using the neighbor-joining method.

FIGURE S2 The nucleotide sequence of the intergeneric spacer region and partial encoding regions of genes 114 and 115. Dotted arrows represent inverted repeats, and an asterisk denotes the stop codon. The detected amino acid residues from MS analysis are underlined. The nucleotide sequence is from position 88621 to 68627 of GenBank Database, accession no. LC373201.2.

TABLE S1 Annotation table of phage EspM4VN.

TABLE S2 General features of EspM4VN and related phages’ genomes.

TABLE S3 MS/MS results for phage proteins identified in Figure 8.
