# Supplementary figures and images for: Characterization and Genome Structure of Virulent Phage EspM4VN to Control Enterobacter sp. M4 Isolated From Plant Soft Rot
Source: Front Microbiol. 2020 Jun 3;11:885. doi: 10.3389/fmicb.2020.00885 (PMC7283392; doi:10.3389/fmicb.2020.00885)

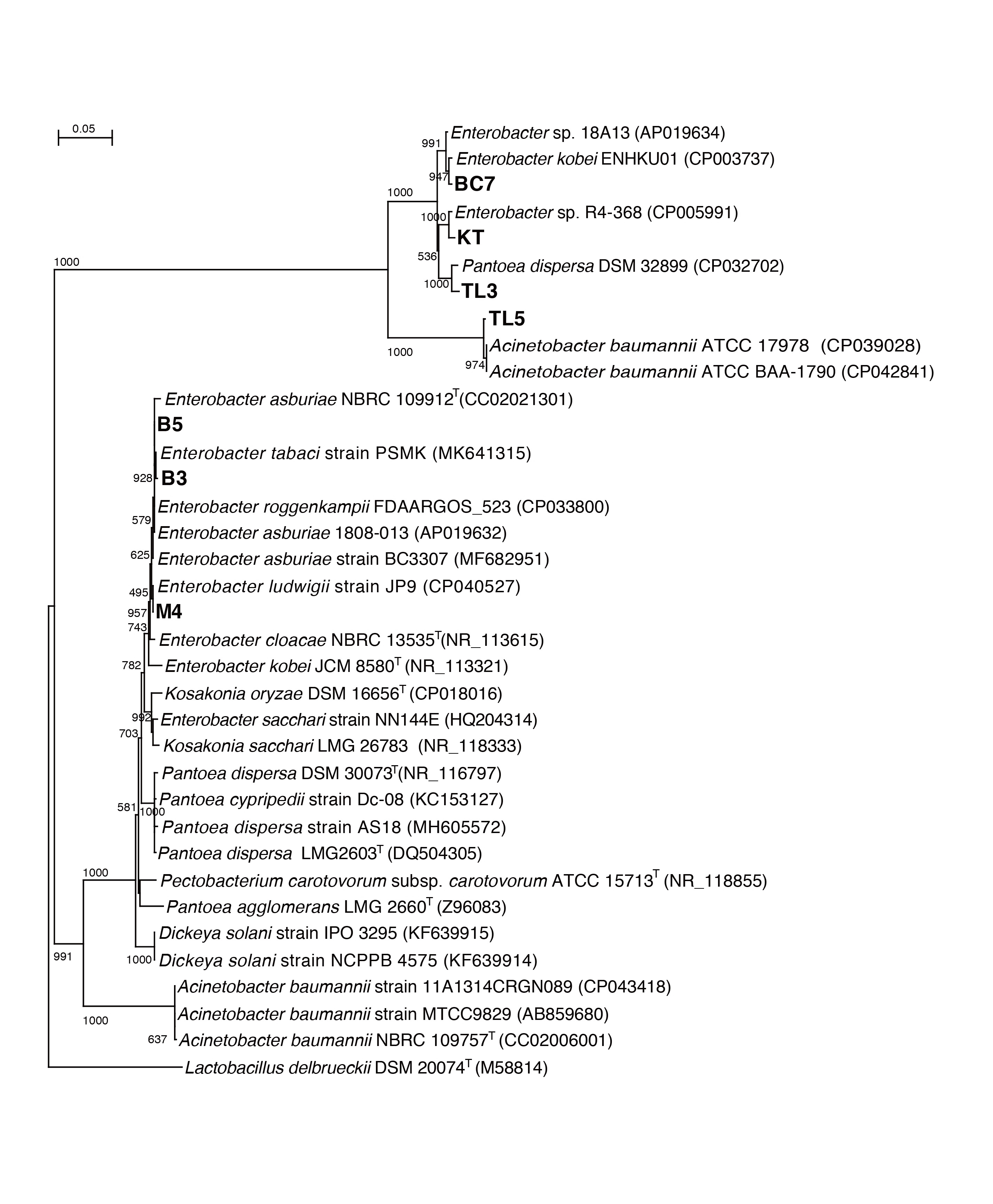

Supplement: FIGURE S1 — Phylogenetic tree of isolated strains. Their nucleotide sequences were compared using ClustalW, and phylogenetic trees were generated using the neighbor-joining method. [file Image_1.jpg]

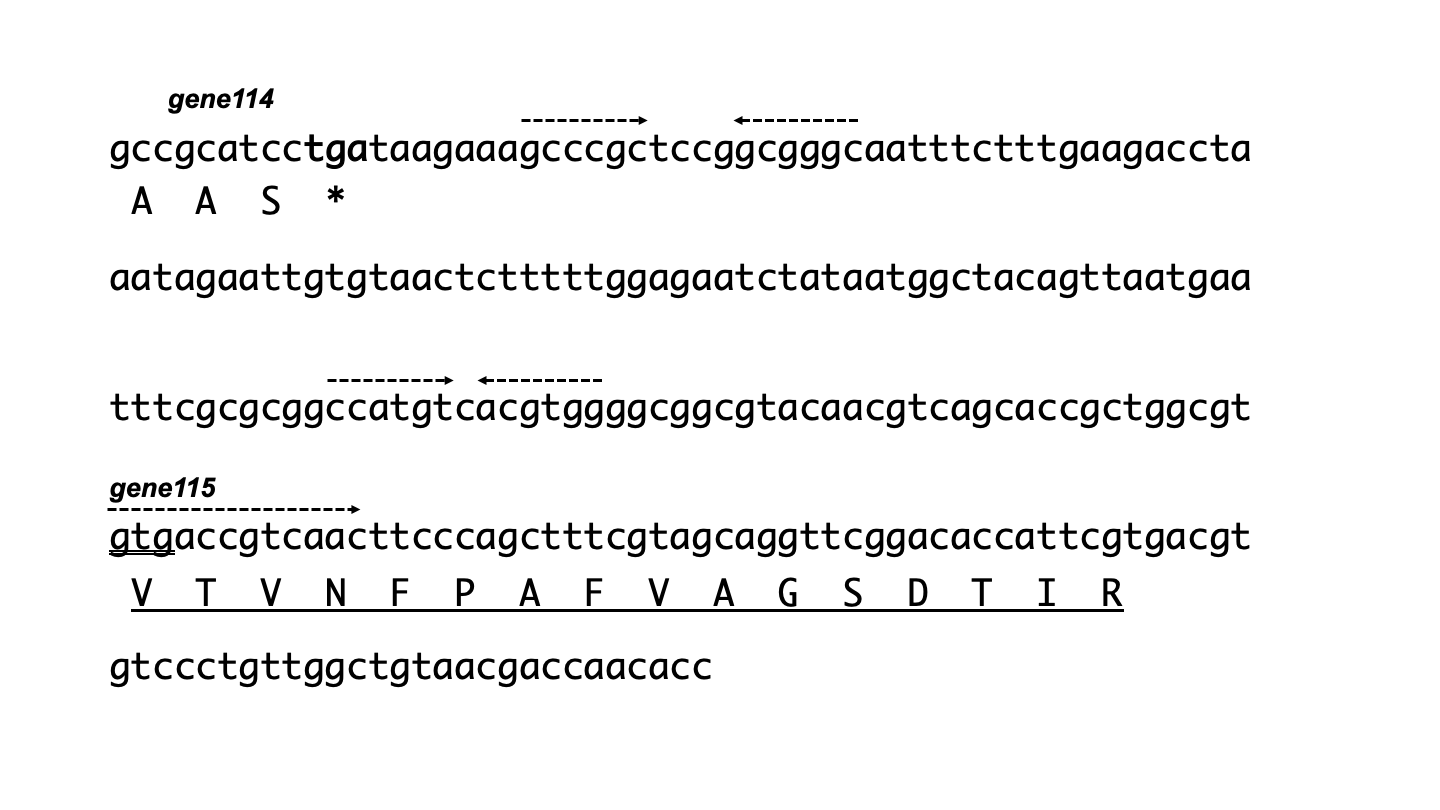

Supplement: FIGURE S2 — The nucleotide sequence of the intergeneric spacer region and partial encoding regions of genes 114 and 115. Dotted arrows represent inverted repeats, and an asterisk denotes the stop codon. The detected amino acid residues from MS analysis are underlined. The nucleotide sequence is from position 88621 to 68627 of GenBank Database, accession no. LC373201.2. [file Image_2.tiff]
