# Supplementary material for: Characterization and Genome Structure of Virulent Phage EspM4VN to Control Enterobacter sp. M4 Isolated From Plant Soft Rot
Source: Front Microbiol. 2020 Jun 3;11:885. doi: 10.3389/fmicb.2020.00885 (PMC7283392; doi:10.3389/fmicb.2020.00885)
Supplement: TABLE S1 — Annotation table of phage EspM4VN. [file Table_1.docx]

**Supplementary Table S1 Annotation table of phage EspM4VN**

| Gene | Start | End | Products | Group | Genbank ID |
| --- | --- | --- | --- | --- | --- |
| gene_1 | 2 | 382 | Hypothetical phage protein | Hypothetical protein | BBD52181.1 |
| gene_2 | 379 | 933 | Putative dUTP diphosphatase | DNA replication | BBD52182.1 |
| gene_3 | 933 | 1475 | Hypothetical protein | Hypothetical protein | BBD52183.1 |
| gene_4 | 1460 | 2551 | RecA recombination protein | DNA replication | BBD52184.1 |
| gene_5 | 2529 | 2858 | Hypothetical protein | Hypothetical protein | BBD52185.1 |
| gene_6 | 2865 | 4292 | Putative DNA primase-helicase subunit gp41 | DNA replication | BBD52186.1 |
| gene_7 | 4355 | 4690 | Hypothetical phage protein | Hypothetical protein | BBD52187.1 |
| gene_8 | 4706 | 5020 | Hypothetical protein | Hypothetical protein | BBD52188.1 |
| gene_9 | 5148 | 6344 | Hypothetical protein | Hypothetical protein | BBD52189.1 |
| gene_10 | 6457 | 6657 | Putative Hypothetical protein | Hypothetical protein | BBD52190.1 |
| gene_11 | 6657 | 6869 | Hypothetical phage protein | Hypothetical protein | BBD52191.1 |
| gene_12 | 6871 | 7218 | Hypothetical phage protein | Hypothetical protein | BBD52192.1 |
| gene_13 | 7278 | 7886 | Putative Hypothetical protein orf00049 | Hypothetical protein | BBD52193.1 |
| gene_14 | 7889 | 8470 | Hypothetical membrane protein | Structure and packaging | BBD52194.1 |
| gene_15 | 8470 | 8655 | Hypothetical protein | Hypothetical protein | BBD52195.1 |
| gene_16 | 8652 | 8831 | Hypothetical protein | Hypothetical protein | BBD52196.1 |
| gene_17 | 8887 | 10314 | DNA ligase | DNA replication | BBD52197.1 |
| gene_18 | 10311 | 10535 | Hypothetical protein | Hypothetical protein | BBD52198.1 |
| gene_19 | 10528 | 10773 | Hypothetical membrane protein | Structure and packaging | BBD52199.1 |
| gene_20 | 10775 | 11032 | Hypothetical membrane protein | Structure and packaging | BBD52200.1 |
| gene_21 | 11073 | 11339 | Hypothetical phage protein | Hypothetical protein | BBD52201.1 |
| gene_22 | 11333 | 11992 | Putative loader of gp41 DNA helicase gp59 | DNA replication | BBD52202.1 |
| gene_23 | 11993 | 13069 | Putative tail length tape measure protein | Structure and packaging | BBD52203.1 |
| gene_24 | 13000 | 13944 | Putative tail length tape measure protein | Structure and packaging | BBD52204.1 |
| gene_25 | 13955 | 15343 | Putative baseplate hub subunit gp27 | Structure and packaging | BBD52205.1 |
| gene_26 | 15340 | 15894 | Putative baseplate wedge subunit gp53 | Structure and packaging | BBD52206.1 |
| gene_27 | 15906 | 16217 | Putative tail tube associated baseplate protein gp48 | Structure and packaging | BBD52207.1 |
| gene_28 | 16924 | 17538 | Gp4 head completion protein | Structure and packaging | BBD52208.1 |
| gene_29 | 17535 | 17747 | Hypothetical protein | Hypothetical protein | BBD52209.1 |
| gene_30 | 17750 | 18157 | Hypothetical protein | Hypothetical protein | BBD52210.1 |
| gene_31 | 18168 | 18674 | Putative deoxycytidylate deaminase | DNA replication | BBD52211.1 |
| gene_32 | 18674 | 18988 | Hypothetical protein | Hypothetical protein | BBD52212.1 |
| gene_33 | 19059 | 19391 | Hypothetical protein | Hypothetical protein | BBD52213.1 |
| gene_34 | 19388 | 19705 | Hypothetical protein | Hypothetical protein | BBD52214.1 |
| gene_35 | 19702 | 20115 | Hypothetical protein | Hypothetical protein | BBD52215.1 |
| gene_36 | 20181 | 20366 | Hypothetical protein | Hypothetical protein | BBD52216.1 |
| gene_37 | 20363 | 20728 | Hypothetical protein | Hypothetical protein | BBD52217.1 |
| gene_38 | 20922 | 21263 | Hypothetical phage protein | Hypothetical protein | BBD52218.1 |
| gene_39 | 21270 | 21965 | Putative Hypothetical protein | Hypothetical protein | BBD52219.1 |
| gene_40 | 21965 | 22450 | Hypothetical protein | Hypothetical protein | BBD52220.1 |
| gene_41 | 22587 | 22892 | Putative Hypothetical protein | Hypothetical protein | BBD52221.1 |
| gene_42 | 22889 | 23521 | Hypothetical protein | Hypothetical protein | BBD52222.1 |
| gene_43 | 23508 | 23903 | Hypothetical protein | Hypothetical protein | BBD52223.1 |
| gene_44 | 23903 | 24502 | Tk.4 protein | DNA replication | BBD52224.1 |
| gene_45 | 24499 | 24741 | Hypothetical protein | Hypothetical protein | BBD52225.1 |
| gene_46 | 24792 | 25202 | Putative Hypothetical protein orf00021 | Hypothetical protein | BBD52226.1 |
| gene_47 | 25206 | 25403 | Hypothetical protein | Hypothetical protein | BBD52227.1 |
| gene_48 | 25623 | 25808 | Hypothetical protein | Hypothetical protein | BBD52228.1 |
| gene_49 | 25851 | 27182 | Putative DNA topoisomerase/gyrase gp52 | DNA replication | BBD52229.1 |
| gene_50 | 27184 | 29097 | Topoisomerase II large subunit | DNA replication | BBD52230.1 |
| gene_51 | 29147 | 29728 | Hypothetical protein | Hypothetical protein | BBD52231.1 |
| gene_52 | 29725 | 30201 | Hypothetical phage protein | Hypothetical protein | BBD52232.1 |
| gene_53 | 30258 | 30455 | Hypothetical protein | Hypothetical protein | BBD52233.1 |
| gene_54 | 30501 | 31001 | Hypothetical protein | Hypothetical protein | BBD52234.1 |
| gene_55 | 31120 | 31356 | Hypothetical protein | Hypothetical protein | BBD52235.1 |
| gene_56 | 31359 | 32168 | Hypothetical protein | Hypothetical protein | BBD52236.1 |
| gene_57 | 32147 | 32539 | Hypothetical protein | Hypothetical protein | BBD52237.1 |
| gene_58 | 32569 | 32775 | Hypothetical protein | Hypothetical protein | BBD52238.1 |
| gene_59 | 32911 | 33264 | Hypothetical protein | Hypothetical protein | BBD52239.1 |
| gene_60 | 33319 | 34881 | Hypothetical protein | Hypothetical protein | BBD52240.1 |
| gene_61 | 34913 | 37669 | RIIA protein | DNA replication | BBD52241.1 |
| gene_62 | 37776 | 38123 | Hypothetical protein | Hypothetical protein | BBD52242.1 |
| gene_63 | 38134 | 38307 | Hypothetical protein | Hypothetical protein | BBD52243.1 |
| gene_64 | 38279 | 38470 | Hypothetical protein | Hypothetical protein | BBD52244.1 |
| gene_65 | 38479 | 39147 | Hypothetical protein | Hypothetical protein | BBD52245.1 |
| gene_66 | 39147 | 39521 | Putative Hypothetical protein orf00256 | Hypothetical protein | BBD52246.1 |
| gene_67 | 39572 | 40018 | Hypothetical protein | Hypothetical protein | BBD52247.1 |
| gene_68 | 40151 | 40720 | Hypothetical protein | Hypothetical protein | BBD52248.1 |
| gene_69 | 40740 | 41855 | Hypothetical protein | Hypothetical protein | BBD52249.1 |
| gene_70 | 41852 | 42238 | Hypothetical protein | Hypothetical protein | BBD52250.1 |
| gene_71 | 42235 | 42459 | Hypothetical protein (Fragment) | Hypothetical protein | BBD52251.1 |
| gene_72 | 42541 | 42708 | Hypothetical protein | Hypothetical protein | BBD52252.1 |
| gene_73 | 42745 | 43980 | Hypothetical protein | Hypothetical protein | BBD52253.1 |
| gene_74 | 43977 | 44162 | Hypothetical membrane protein | Structure and packaging | BBD52254.1 |
| gene_75 | 44220 | 44516 | Hypothetical protein | Hypothetical protein | BBD52255.1 |
| gene_76 | 44513 | 44710 | Hypothetical protein | Hypothetical protein | BBD52256.1 |
| gene_77 | 44713 | 45627 | Hypothetical protein | Hypothetical protein | BBD52257.1 |
| gene_78 | 45627 | 45932 | Hypothetical phage protein | Hypothetical protein | BBD52258.1 |
| gene_79 | 45942 | 46520 | Putative 5'(3') deoxyribonucleotidase | Signal transduction and regulatory | BBD52259.1 |
| gene_80 | 46490 | 46717 | Hypothetical protein | Hypothetical protein | BBD52260.1 |
| gene_81 | 46714 | 47055 | Hypothetical phage protein | Hypothetical protein | BBD52261.1 |
| gene_82 | 47117 | 50116 | DNA polymerase | DNA replication | BBD52262.1 |
| gene_83 | 50196 | 50768 | Hypothetical protein | Hypothetical protein | BBD52263.1 |
| gene_84 | 50811 | 51023 | Hypothetical protein | Hypothetical protein | BBD52264.1 |
| gene_85 | 51119 | 51646 | Hypothetical protein | Hypothetical protein | BBD52265.1 |
| gene_86 | 51695 | 52246 | Hypothetical protein | Hypothetical protein | BBD52266.1 |
| gene_87 | 52246 | 52605 | Hypothetical protein | Hypothetical protein | BBD52267.1 |
| gene_88 | 52631 | 53089 | Hypothetical protein | Hypothetical protein | BBD52268.1 |
| gene_89 | 53100 | 53588 | Hypothetical protein | Hypothetical protein | BBD52269.1 |
| gene_90 | 53628 | 54821 | Hypothetical protein | Hypothetical protein | BBD52270.1 |
| gene_91 | 54891 | 55592 | Hypothetical phage protein | Hypothetical protein | BBD52271.1 |
| gene_92 | 55663 | 56181 | Hypothetical protein | Hypothetical protein | BBD52272.1 |
| gene_93 | 56190 | 56426 | Hypothetical protein | Hypothetical protein | BBD52273.1 |
| tRNA | 56908 | 56996 | tRNA-Ser |  |  |
| tRNA | 57256 | 57339 | tRNA-Tyr |  |  |
| tRNA | 57597 | 57672 | tRNA-Asn |  |  |
| gene_94 | 57674 | 57871 | Hypothetical protein | Hypothetical protein | BBD52274.1 |
| gene_95 | 57908 | 58135 | Hypothetical protein | Hypothetical protein | BBD52275.1 |
| tRNA | 58560 | 58639 | tRNA-Ser |  |  |
| gene_96 | 58921 | 59133 | Hypothetical protein | Hypothetical protein | BBD52276.1 |
| gene_97 | 60617 | 61189 | Hypothetical phage protein | Hypothetical protein | BBD52277.1 |
| gene_98 | 61538 | 63316 | Putative baseplate wedge subunit gp6 | Structure and packaging | BBD52278.1 |
| gene_99 | 63300 | 64154 | Putative baseplate wedge subunit gp7 | Structure and packaging | BBD52279.1 |
| gene_100 | 64157 | 65368 | Hypothetical protein | Hypothetical protein | BBD52280.1 |
| gene_101 | 65421 | 68750 | Hypothetical protein | Hypothetical protein | BBD52281.1 |
| gene_102 | 68796 | 70592 | Hypothetical protein | Hypothetical protein | BBD52282.1 |
| gene_103 | 70647 | 73211 | Hypothetical protein | Hypothetical protein | BBD52283.1 |
| gene_104 | 73276 | 75846 | Hypothetical protein | Hypothetical protein | BBD52284.1 |
| gene_105 | 75943 | 80703 | Hypothetical protein | Hypothetical protein | BBD52285.1 |
| gene_106 | 80832 | 81083 | Uncharacterzed protein | Hypothetical protein | BBD52286.1 |
| gene_107 | 81064 | 81402 | Hypothetical protein | Hypothetical protein | BBD52287.1 |
| gene_108 | 81392 | 82144 | Hypothetical protein | Hypothetical protein | BBD52288.1 |
| gene_109 | 82172 | 82384 | Hypothetical protein | Hypothetical protein | BBD52289.1 |
| gene_110 | 82444 | 83094 | Putative neck protein gp14 | Structure and packaging | BBD52290.1 |
| gene_111 | 83097 | 83792 | Putative tail sheath stabilizer gp15 | Structure and packaging | BBD52291.1 |
| gene_112 | 83795 | 84490 | Putative terminase small subunit gp16 | Structure and packaging | BBD52292.1 |
| gene_113 | 84471 | 86681 | Gp17 terminase DNA packaging enzyme large subunit | Structure and packaging | BBD52293.1 |
| tRNA | 84879 | 84966 | tRNA-Asp |  |  |
| gene_114 | 86734 | 88632 | Putative tail sheath protein gp18 | Structure and packaging | BBD52294.1 |
| gene_115 | 88780 | 89241 | Putative tail tube protein gp19 | Structure and packaging | BBD52295.1 |
| gene_116 | 89309 | 91000 | Putative portal protein | Structure and packaging | BBD52296.1 |
| gene_117 | 91039 | 91206 | Hypothetical protein | Hypothetical protein | BBD52297.1 |
| gene_118 | 91217 | 91522 | Hypothetical protein | Hypothetical protein | BBD52298.1 |
| gene_119 | 91533 | 92198 | Putative prohead protease gp21 | Structure and packaging | BBD52299.1 |
| gene_120 | 92244 | 93119 | Gp22 prohead core protein | Structure and packaging | BBD52300.1 |
| gene_121 | 93211 | 94533 | Gp23 major head protein | Structure and packaging | BBD52301.1 |
| gene_122 | 94626 | 94850 | Hypothetical protein | Hypothetical protein | BBD52302.1 |
| gene_123 | 94943 | 95260 | Putative Hypothetical protein | Hypothetical protein | BBD52303.1 |
| gene_124 | 95318 | 96175 | Hypothetical protein | Hypothetical protein | BBD52304.1 |
| gene_125 | 96233 | 96457 | Hypothetical protein | Hypothetical protein | BBD52305.1 |
| gene_126 | 96475 | 96915 | Hypothetical protein | Hypothetical protein | BBD52306.1 |
| gene_127 | 96924 | 97160 | Hypothetical phage protein | Hypothetical protein | BBD52307.1 |
| gene_128 | 97264 | 97560 | Hypothetical protein | Hypothetical protein | BBD52308.1 |
| gene_129 | 97630 | 97950 | Hypothetical phage protein | Hypothetical protein | BBD52309.1 |
| gene_130 | 97956 | 98378 | Hypothetical protein | Hypothetical protein | BBD52310.1 |
| gene_131 | 98420 | 98587 | Hypothetical protein | Hypothetical protein | BBD52311.1 |
| gene_132 | 98625 | 99353 | Hypothetical protein | Hypothetical protein | BBD52312.1 |
| gene_133 | 99354 | 99878 | Hypothetical protein | Hypothetical protein | BBD52313.1 |
| gene_134 | 100034 | 100534 | Putative tail completion & sheath stabilizer protein gp3 | Structure and packaging | BBD52314.1 |
| gene_135 | 100577 | 101029 | Putative DNA repair/recombination protein UvsY | DNA replication | BBD52315.1 |
| gene_136 | 101029 | 101775 | Putative exonuclease | Signal transduction and regulatory | BBD52316.1 |
| gene_137 | 101804 | 101977 | Hypothetical protein | Hypothetical protein | BBD52317.1 |
| gene_138 | 101953 | 103311 | RNA-DNA and DNA-DNA helicase UvsW | DNA replication | BBD52318.1 |
| gene_139 | 103302 | 103678 | Hypothetical protein | Hypothetical protein | BBD52319.1 |
| gene_140 | 104024 | 104692 | Sliding clamp DNA polymerase accessory protein | DNA replication | BBD52320.1 |
| gene_141 | 104772 | 105761 | Putative DNA polymerase accessory protein gp44 | DNA replication | BBD52321.1 |
| gene_142 | 105766 | 106188 | Putative clamp loader subunit gp62 | Signal transduction and regulatory | BBD52322.1 |
| gene_143 | 106218 | 106682 | Hypothetical protein | Hypothetical protein | BBD52323.1 |
| gene_144 | 106699 | 107562 | Nucleoside triphosphate pyrophosphohydrolase | Signal transduction and regulatory | BBD52324.1 |
| gene_145 | 107631 | 108845 | Hypothetical protein | Hypothetical protein | BBD52325.1 |
| gene_146 | 108961 | 110991 | Hypothetical protein | Hypothetical protein | BBD52326.1 |
| gene_147 | 111036 | 111407 | Hypothetical protein | Hypothetical protein | BBD52327.1 |
| gene_148 | 111849 | 112643 | Hypothetical protein | Hypothetical protein | BBD52328.1 |
| gene_149 | 112754 | 113608 | Ribose-phosphate pyrophosphokinase | DNA replication | BBD52329.1 |
| gene_150 | 113605 | 115269 | Nicotinamide phosphoribosyltransferase | DNA replication | BBD52330.1 |
| gene_151 | 115311 | 115481 | Hypothetical phage protein | Hypothetical protein | BBD52331.1 |
| gene_152 | 115474 | 117705 | Putative vWa containing protein | DNA replication | BBD52332.1 |
| gene_153 | 117748 | 118074 | Hypothetical protein | Hypothetical protein | BBD52333.1 |
| gene_154 | 118198 | 118437 | Hypothetical protein | Hypothetical protein | BBD52334.1 |
| gene_155 | 118437 | 118682 | Hypothetical protein | Hypothetical protein | BBD52335.1 |
| gene_156 | 118742 | 119197 | Putative pyimidine dimer DNA glycosylase DenV | DNA replication | BBD52336.1 |
| gene_157 | 119210 | 119584 | Hypothetical protein | Hypothetical protein | BBD52337.1 |
| gene_158 | 119581 | 119868 | Hypothetical protein | Hypothetical protein | BBD52338.1 |
| gene_159 | 119870 | 120028 | Hypothetical protein | Hypothetical protein | BBD52339.1 |
| gene_160 | 120062 | 120511 | Hypothetical protein | Hypothetical protein | BBD52340.1 |
| gene_161 | 120614 | 121276 | Hypothetical protein | Hypothetical protein | BBD52341.1 |
| gene_162 | 121273 | 121605 | Hypothetical protein | Hypothetical protein | BBD52342.1 |
| gene_163 | 121655 | 122581 | Hypothetical phage protein | Hypothetical protein | BBD52343.1 |
| gene_164 | 122638 | 123096 | Hypothetical protein | Hypothetical protein | BBD52344.1 |
| gene_165 | 123029 | 123289 | Hypothetical phage protein | Hypothetical protein | BBD52345.1 |
| gene_166 | 123291 | 123740 | Hypothetical protein | Hypothetical protein | BBD52346.1 |
| gene_167 | 123833 | 124111 | Putative DNA-binding protein | Signal transduction and regulatory | BBD52347.1 |
| gene_168 | 124241 | 124585 | Hypothetical protein | Hypothetical protein | BBD52348.1 |
| gene_169 | 124471 | 125958 | Putative ATP-dependent DNA helicase | DNA replication | BBD52349.1 |
| gene_170 | 125955 | 126722 | Hypothetical protein | Hypothetical protein | BBD52350.1 |
| gene_171 | 126768 | 127295 | Putative ribonuclease HI | DNA replication | BBD52351.1 |
| gene_172 | 127307 | 128098 | Gp55 T4-like sigma factor involved in late transcription | Signal transduction and regulatory | BBD52352.1 |
| gene_173 | 128085 | 129200 | Putative endonuclease gp47 | Signal transduction and regulatory | BBD52353.1 |
| gene_174 | 129203 | 131539 | Putative recombination endonuclease subunit gp46 | DNA replication | BBD52354.1 |
| gene_175 | 131542 | 131706 | Hypothetical protein | Hypothetical protein | BBD52355.1 |
| gene_176 | 131703 | 132032 | Hypothetical protein | Hypothetical protein | BBD52356.1 |
| gene_177 | 132013 | 132297 | Hypothetical protein | Hypothetical protein | BBD52357.1 |
| gene_178 | 132397 | 132397 | Putative RegB protein | Signal transduction and regulatory | BBD52358.1 |
| gene_179 | 133011 | 133649 | Hypothetical protein | Hypothetical protein | BBD52359.1 |
| gene_180 | 133757 | 134128 | Hypothetical protein | Hypothetical protein | BBD52360.1 |
| gene_181 | 134151 | 134462 | Hypothetical protein | Hypothetical protein | BBD52361.1 |
| gene_182 | 134536 | 136176 | Hypothetical protein | Hypothetical protein | BBD52362.1 |
| gene_183 | 136233 | 136802 | Hypothetical protein | Hypothetical protein | BBD52363.1 |
| gene_184 | 136812 | 137294 | Hypothetical protein | Hypothetical protein | BBD52364.1 |
| gene_185 | 137345 | 137698 | Hypothetical protein | Hypothetical protein | BBD52365.1 |
| gene_186 | 137758 | 138267 | Hypothetical protein | Hypothetical protein | BBD52366.1 |
| gene_187 | 138267 | 138878 | Hypothetical protein | Hypothetical protein | BBD52367.1 |
| gene_188 | 138878 | 139936 | DNA primase | DNA replication | BBD52368.1 |
| gene_189 | 139933 | 140139 | Hypothetical phage protein | Hypothetical protein | BBD52369.1 |
| gene_190 | 140335 | 140823 | Hypothetical protein | Hypothetical protein | BBD52370.1 |
| gene_191 | 140885 | 141073 | Hypothetical protein | Hypothetical protein | BBD52371.1 |
| gene_192 | 141070 | 141357 | Hypothetical protein | Hypothetical protein | BBD52372.1 |
| gene_193 | 141427 | 142221 | Putative endolysin | Endolysin | BBD52373.1 |
| gene_194 | 142326 | 143168 | PhoH-like protein | Signal transduction and regulatory | BBD52374.1 |
| gene_195 | 143254 | 145533 | Ribonucleoside-diphosphate reductase | DNA replication | BBD52375.1 |
| gene_196 | 145604 | 146707 | NrdB ribonucleotide reductase subunit beta | DNA replication | BBD52376.1 |
| gene_197 | 146717 | 146941 | Hypothetical protein | Hypothetical protein | BBD52377.1 |
| gene_198 | 147047 | 147508 | Hypothetical protein | Hypothetical protein | BBD52378.1 |
| gene_199 | 147515 | 147904 | Hypothetical protein | Hypothetical protein | BBD52379.1 |
| gene_200 | 147905 | 148285 | Hypothetical protein | Hypothetical protein | BBD52380.1 |
| gene_201 | 148358 | 149968 | Gp5 baseplate hub subunit and tail lysozyme | Structure and packaging | BBD52381.1 |
| gene_202 | 150479 | 151183 | Putative baseplate hub subunit gp26 | Structure and packaging | BBD52382.1 |
| gene_203 | 151336 | 151860 | Hypothetical protein | Hypothetical protein | BBD52383.1 |
| gene_204 | 151838 | 152329 | Hypothetical protein | Hypothetical protein | BBD52384.1 |
| gene_205 | 152369 | 152971 | Putative holliday junction resolvase RuvC | DNA replication | BBD52385.1 |
| gene_206 | 153505 | 153750 | Putative late promoter transcription accessory protein gp33 | Signal transduction and regulatory | BBD52386.1 |
| gene_207 | 153758 | 153997 | Hypothetical protein | Hypothetical protein | BBD52387.1 |
| gene_208 | 154096 | 155142 | Putative ssDNA binding protein gp32 | Signal transduction and regulatory | BBD52388.1 |
| gene_209 | 155169 | 155501 | Putative baseplate tail tube initiator gp54 | Structure and packaging | BBD52389.1 |
| gene_210 | 156165 | 156542 | Putative DNA end protector protein gp2 | Signal transduction and regulatory | BBD52390.1 |
| gene_211 | 156473 | 156865 | Gp2 DNA end protector protein | Signal transduction and regulatory | BBD52391.1 |
| gene_212 | 156931 | 157095 | Hypothetical protein | Hypothetical protein | BBD52392.1 |
| gene_213 | 157092 | 157682 | Hypothetical protein | Hypothetical protein | BBD52393.1 |
| gene_214 | 157889 | 158557 | Hypothetical protein | Hypothetical protein | BBD52394.1 |
| gene_215 | 158476 | 158814 | Hypothetical protein | Hypothetical protein | BBD52395.1 |
| gene_216 | 159178 | 159564 | Hypothetical protein | Hypothetical protein | BBD52396.1 |
| gene_217 | 159805 | 160071 | Thymidylate synthase | DNA replication | BBD52397.1 |
| gene_218 | 160029 | 160370 | Putative thymidylate synthase | DNA replication | BBD52398.1 |
| gene_219 | 160354 | 160515 | Thymidylate synthase | DNA replication | BBD52399.1 |
