# Supplementary material for: Characterization and Genome Structure of Virulent Phage EspM4VN to Control Enterobacter sp. M4 Isolated From Plant Soft Rot
Source: Front Microbiol. 2020 Jun 3;11:885. doi: 10.3389/fmicb.2020.00885 (PMC7283392; doi:10.3389/fmicb.2020.00885)
Supplement: TABLE S2 — General features of EspM4VN and related phages’ genomes. [file Table_2.docx]

**Supplementary Table S2 General features of EspM4VN and related phages’ genomes**

| **Features** | **Bacteriophages** | | | | | | | | | | | |
| --- | --- | --- | --- | --- | --- | --- | --- | --- | --- | --- | --- | --- |
|  | **EspM4VN** | **SboM-AG3** | **SKML-39** | **SH19** | **Coodle** | **PP35** | **JA15** | **LIMEstone1** | **phiDP23.1** | **D3** | **D5** | **XF4** |
| **Genome size (bp)** | 160,766 | 158,006 | 159,624 | 157,785 | 152,515 | 152,048 | 153,757 | 152,427 | 188,540 | 152 308 | 155,346 | 151519 |
| **% of coding regions in the genome** | 90.6 | 92.7 | 88.6 | - | 94.4 | 92.7 | 90.7 | - | 83.2 | 91.2 | 89.9 | 91 |
| **Number of predicted ORFs (PEGs)** | 219 | 216 | 209 | 339 | 202 | 198 | 199 | 201 | 223 | 191 | 196 | 196 |
| **% of PEGs with assigned fuctions** | 30% with assigned 9% with hypthetical functions 61% with unknown function | 33% with assigned functions 67% with hypothetical functions | 9% with assigned functions 91% with hypothetical functions | 31% with assigned functions 17% with hypothetical functions 52% with unknown function | 42% with assigned functions 58% with hypothetical functions | 54% with assigned functions 46% with hypothetical functions | 45% with assigned 48% with hypthetical functions 7% with unknown function | 46% with assigned 54% with unknown function | 39.5% with assigned 22.4% with cal functions 8% with unknown function | 54.9% with assigned 45.1% with hypthetical functions | 25.5% with assigned 49% with hypthetical functions 25.5% with unknown function |  |
| **Average gene length (bp)** | 653 | 731 | 677 | - | 709 | 712 | 693 | - | 538-804 | 730 | 711 | 703 |
| **Number of PEGs in functional groups** | Phage replication (8)  Phage tail proteins 2 (1)  Phage lysis modules (1) Nucleosides and Nucleotides (2) | - | Phage tail proteins 2 (1)  Phage tail fiber proteins (1)  Phage lysis modules (1) RNA Metabolism (1) Nucleosides and Nucleotides (2)  DNA Metabolism (1) | Phage replication (10)  Phage tail proteins 2 (1)  Phage lysis modules (1) Nucleosides and Nucleotides (2) | Phage replication (9)  Phage tail proteins 2 (1)  Phage lysis modules (1) RNA Metabolism (1) Nucleosides and Nucleotides (2) DNA Metabolism (1) | Phage replication (14)  Phage tail proteins 2 (1)  Phage tail fiber proteins (1)  Phage lysis modules (1) RNA processing and modification (1) Nucleosides and Nucleotides (2) DNA Metabolism (1) | Phage replication (14)  Phage tail proteins 2 (1)  Phage tail fiber proteins (1)  Phage lysis modules (1)  RNA Metabolism (1) Nucleosides and Nucleotides (2) DNA Metabolism (1) | Phage replication (10)  Phage tail proteins 2 (1)  Phage lysis modules (1) RNA Metabolism (1) Nucleosides and Nucleotides (2) DNA Metabolism (1) | - | Phage replication (10)  Phage tail proteins 2 (1)  Phage lysis modules (1) RNA Metabolism (1)  Nucleosides and Nucleotides (2) DNA Metabolism (1) | - | Phage replication (14)  Phage tail proteins 2 (1)  Phage tail fiber proteins (1)  Phage lysis modules (1) RNA metablolism (1) Nucleosides and Nucleotides (2) DNA Metabolism (1) |
| **Transcription start codon (% of genes of this start codon)** | - | ATG (95.4%) GTG (3.2%) CTG (0.9%) TTG (0.5%) | - | - | - | - | - | - | ATG (85.3%) GTG (13.6%) TTG (1.1%) | - | ATG (94.4%) GTG (4.1%) TTG (1.5%) | - |
